# Analysis of the Unique Historical Isolate of African Swine Fever Virus Isolate Spencer from Outbreaks in 1951

**DOI:** 10.3390/v16081175

**Published:** 2024-07-23

**Authors:** Edward Spinard, Mark Dinhobl, Jacob Fenster, Charronne Davis, Manuel V. Borca, Douglas P. Gladue

**Affiliations:** 1 Plum Island Animal Disease Center (PIADC), ARS, USDA, P.O. Box 848, Greenport, NY 11944, USA; edward.spinard@usda.gov (E.S.); mark.dinhobl@usda.gov (M.D.); jacob.fenster@usda.gov (J.F.); 2 National Bio and Agro-Defense Facility, Foreign Animal Disease Research Unit, Agricultural Research Service, U.S. Department of Agriculture, Manhattan, KS 66502, USA; 3 Oak Ridge Institute for Science and Education (ORISE), Oak Ridge, TN 37830, USA; 4 American Type Culture Collection, 0801 University Blvd, Manassas, VA 20108, USA; cdavis@atcc.org

**Keywords:** ASFV, South Africa, 1951, African swine fever, African swine fever virus

## Abstract

African swine fever (ASF) is a deadly hemorrhagic disease of domestic and wild swine that was first described in the early 20th century after the introduction of European pigs to Kenya. The etiological agent, the African swine fever virus (ASFV), is a large DNA virus within the *Asfarviridae* family that is broadly categorized epidemiologically into genotypes based on the nucleotide sequence of B646L, the gene encoding the major capsid protein p72. ASF outbreaks in Africa have been linked historically to 25 genotypes by p72 nucleotide analysis and, recently, to 6 genotypes by amino acid comparison, whereas global outbreaks of ASF outside of Africa have only been linked to 2 genotypes: genotype I, which led to an outbreak in Europe during the 1960s that later spread to South America, and genotype II, responsible for the current pandemic that began in Georgia in 2007 and has since spread to Europe, Asia, and Hispaniola. Here, we present an analysis of the genome of ASFV Spencer, an isolate that was collected in 1951 near Johannesburg, South Africa. While nucleotide analysis of Spencer indicates the p72 coding sequence is unique, differentiating from the closest reference by five nucleotides, the predicted amino acid sequence indicates that it is 100% homologous to contemporary genotype 1. Full genome analysis reveals it is more similar to Mkuzi1979 and encodes genes that share similarity with either genotype 1 or genotype 2 outbreak strains.

## 1. Introduction

Currently, African swine fever (ASF) is circulating and causing repeated outbreaks throughout Africa, Asia, Europe, and the island of Hispaniola. ASF is caused by a large double-stranded virus, the African swine fever virus (ASFV), which encodes for over 180 different proteins and is typically over 190 kbp in length. Before the widespread use of next-generation sequencers (NGSs), ASFV was very difficult to sequence. Monitoring outbreaks and the spread of the disease was limited to small PCR-amplified sequences that were then sequenced by Sanger sequencing. Very few genomes of the ASFV are publicly available, with most of the publicly available genomes belonging to recent outbreaks, particularly of genotype 1 and genotype 2, as characterized by the sequence of p72.

Recently, we reported on the re-analysis of ASFV genotyping using the protein sequence of p72 [1], as genotyping of a fragment of p72 at the nucleotide level was done historically out of convenience and limitations of sequencing. However, there was confusion among labs, and data entry errors became incorporated, ultimately resulting in the incorrect assignment of genotypes. The method of p72 genotyping has no biological significance; however, genotyping by using the predicted amino acid sequence of full-length p72 allows for direct comparison at a potential functional level among isolates of one of the major capsid proteins of the ASFV. Accordingly, we performed a back-analysis using all open reading frames of the ASFV [2]. Upon examination of the entire proteome, as opposed to a single gene, a number of African swine fever isolates were found to be reclassified despite having identical p72 proteins. This reassignment occurred due to the fact that these isolates exhibited a closer genomic similarity to other isolates when considering the entire genome rather than just the p72 protein.

Analyzing historical isolates through sequencing presents a unique opportunity to delve into the past and explore the epidemiological history of the ASFV. By analyzing isolates gathered from Madagascar, Mozambique, and Mauritius, which precede the 2007 outbreak in Georgia, researchers have uncovered the probable source of the present pandemic strain [3]. The prevailing belief is that the 1957 genotype I outbreak in Portugal originated in Angola. Nevertheless, a thorough examination of the K49 isolate, obtained from the Democratic Republic of the Congo in 1949, demonstrated the presence of a genotype I strain, indicating that genotype I was not limited solely to western Africa [4]. Despite their value, since samples are restricted to specific years and regions, historical isolates can only offer a limited perspective on the past. It is true that even today, there is evidence to suggest that outbreaks within the same country in Africa can result from different strains. Still, studying historical isolates has the potential to improve our understanding of ASFV evolution and could be utilized as a point of comparison for emerging genomes.

Here, we present the fully sequenced genome of ASFV Spencer. In 1951, ASFV Spencer was isolated from the blood of a pig that displayed typical symptoms of African swine fever on a farm near Johannesburg, South Africa [5]. An aliquot of this isolate has been maintained at the Plum Island Animal Disease Center since its collection, but additional details are not certain, as records were not maintained historically until 1951, and only the limited information of pig blood from a farm near Johannesburg in South Africa is available. However, the unique sequence of this isolate is of historical importance as we start to understand the evolution of ASFV isolates. An analysis of the full p72 coding region demonstrates that Spencer differs from the most similar sequence by five nucleotides. An analysis of the partial C-terminal region indicates that it differs from genotype I isolates by two nucleotides. Further, the predicted amino acid sequence of p72 is 100% homologous to genotype 1 isolates. An analysis of the full genome sequence reveals that Spencer is most closely related to Mkuzi1979 (accession AY261362), an isolate collected from South Africa in 1979 from a tick. Mkuzi1979 is classified as historical genotype VII (partial p72 nucleotide genotyping) and genotype I (genotyping by full-length p72 amino acids). Further, an analysis of the predicted amino acid sequences of the open reading frames (ORFs) indicates that some ORFs were more similar to the prototypical genotype II isolate ASFV Georgia 2007/1 (FR68f2468) than the genotype I isolate L60 (KM262844) or K49 (MZ202520).

## 2. Materials and Methods

### 2.1. Next-Generation Sequencing of ASFV Spencer

Virus DNA from the infected macrophage cultures that showed 90–100% CPE was obtained using the Nuclear Extract Kit (Active Motif, Carlsbad, CA, USA). After separation from the nucleus, the cytoplasmic fraction was used to obtain the viral DNA by following the manufacturer’s protocol. Briefly, virus-infected cells were harvested and treated with the hypotonic buffer on ice for 15 min (or until the cell membrane was dissolved). Then, the fraction containing the nucleus was separated by centrifugation, the cytoplasmic fraction was collected, and the DNA was collected using a virus DNA isolation kit (Qiagen, Hilden, Germany) per the manufacturer’s instructions. These were then centrifuged at max speed in a tabletop centrifuge. Then, the aqueous phase was precipitated using 2 volumes of 100% ethanol, washed with the same volume of 70% ethanol, and dried. The obtained pellet of DNA was then resuspended in sterile water. The DNA library was then used for NGS sequencing using the Nextera XT kit in the NextSeq sequencer (Illumina, San Diego, CA, USA), strictly following the manufacturer’s protocol.

### 2.2. De Novo Assembly of ASFV Spencer

Illumina reads were trimmed using fastP [6] to remove adapters, ambiguous nucleotides (with a maximum of 2), and to ensure a minimum length of 50 nucleotides. Additionally, quality filtering was applied with a minimum Phred score of 20, and 25 nucleotides were removed from the 5′ end and 5 nucleotides from the 3′ end. This trimming process resulted in approximately 56 million paired reads. To eliminate host reads, the reads that aligned to Georgia 2007/1 (FR682468) [7] using the default parameters of bwa-mem2 [8] were collected. The remaining reads were then mapped to the *Sus scrofa* genome [9] using the default parameters of bwa-mem2 except for K = 45, and the reads that did not map were collected. For de novo assembly, SPAdes [10] was used with the kmers set to 21, 33, 55, 75, and 99, resulting in a 187,717-nucleotide length contig with a GC content of 38.5%. To determine coverage, trimmed Illumina reads were aligned to the final contig using bwa-mem2, resulting in 497 × coverage.

### 2.3. Annotation

The genome was annotated using the default settings of TheTransporter [11] against a curated collection of ASFV CDS nucleotide sequences, resulting in the annotation of 166 ORFs.

### 2.4. Sequence Alignment and Phylogenic Trees

The Spencer genome was aligned to BA71V (U18466), Benin 97/1 (AM712239), OURT_88_3 (AM712240), Kenya_1950 (AY261360), Malawi_Lil_20_1 (AY261361), Mkuzi1979 (AY261362), Pret_96_4 (AY261363), Tengani62 (AY261364), Warmbaths (AY261365), Warthog (AY261366), E75 (FN557520), Ken05 (KM111294), Ken06Bus (KM111295), L60 (KM262844), NHV (KM262845), K49 (MZ202520), and Georgia 2007/1 (LR743116) using the Whole Genome Alignment tool of CLC Genomics Workbench v23 (Qiagen). From this alignment, the Create Average Nucleotide Identity tool (min similarity fraction and min length fraction = 0.8) was used to calculate the average nucleotide identity, followed by Create Tree from Comparison (Unweighted Pair Group Method with Arithmetic Mean (UPGMA)) to create a phylogenic tree.

ORFs originating from Spencer, Mkuzi1979, Georgia2007/1, L60, and K49 were translated using CLC Genomic Workbench’s “Translate to Protein” and aligned using MUSCLE [12], using the following default parameters: Gap Open = 10, Gap extension = 1. Protein alignment files were imported into CLC Genomics Workbench, and “Create Pairwise Comparisons” was performed to create matrices that detailed the number of differences, percent identity, and distances (Jukes–Cantor) between ORF homologs encoded by the different isolates.

## 3. Results and Discussion

ASFV Spencer was sequenced and assembled using the ASFV pipeline described in the Materials and Methods section. Though only short-length Illumina reads were used, a single 187,717-nucleotide contig was assembled.

### 3.1. Genotyping of Spencer

**Figure 1:**
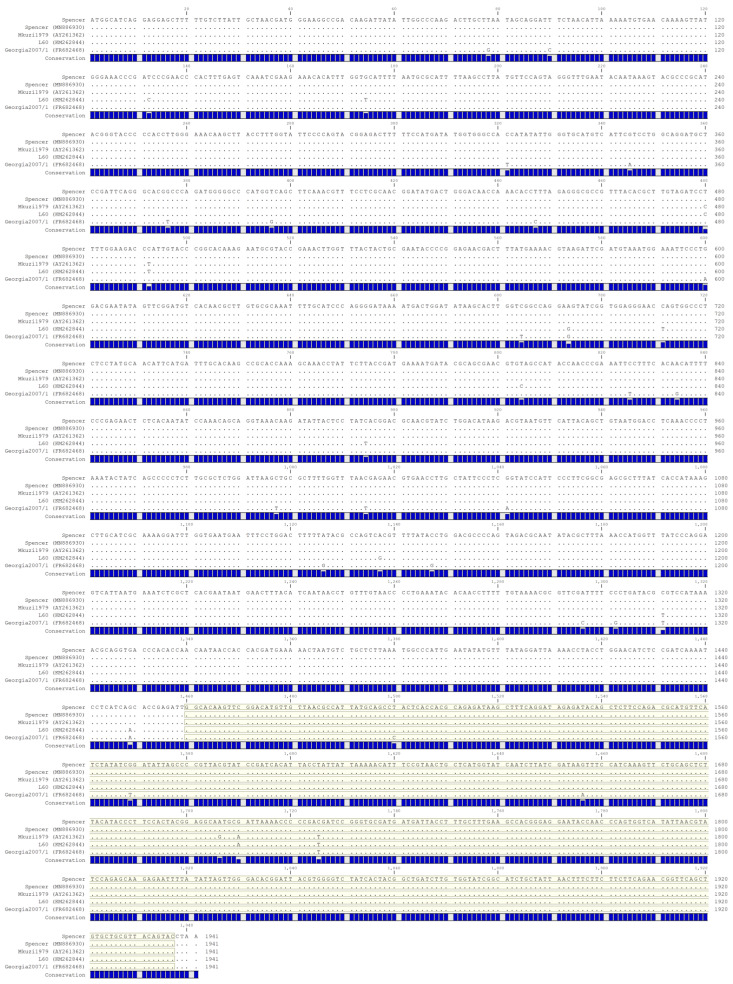
Nucleotide alignment of the p72 coding region. The region highlighted in yellow indicates the 478 C-terminal region used in the historic genotype classification. A royal blue bar graph beneath each sequence indicates nucleotide conservation.

**Figure 2:**
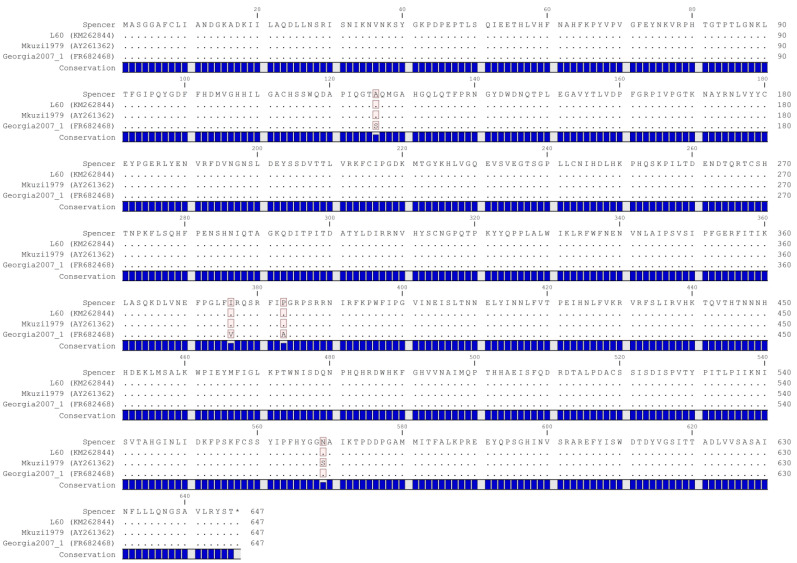
Predicted amino acid alignment of the p72 coding region. Amino acid variations are highlighted. A royal blue bar graph beneath each sequence indicates nucleotide conservation.

Genotyping was performed based on the nucleotide and predicted amino acid sequence of p72 (B646L) encoded by Spencer. Interestingly, an analysis of the full-length B646L nucleotide sequence indicates that this sequence encoded by Spencer is unique. When compared to the NCBI database via blastn, the only identical sequence identified was itself, as it had previously been sequenced via Sanger sequencing (accession MN886930) [13]. Further, the full-length B646L encoded by Spencer differs from the most similar sequence, encoded by Mkuzi1979, by five nucleotides (Figure 1). Within the partial C-terminal region, Spencer differs by three (Mkuzi1979) or two nucleotides (L60 and Georgia 2007/1) from the examined strains (Figure 1, highlighted box). Even though the full-length p72 nucleotide sequence is most similar to Mkuzi1979, the predicted amino acid sequence encoded by Spencer is not 100% identical to Mkuzi1979, as one of the SNPs results in a change in amino acid sequence that is unique to Mkuzi1979 (Figure 2). Accordingly, as Spencer’s p72 predicted amino acid sequence was 100% homologous to L60, Spencer belongs to genotype 1.

### 3.2. Whole-Genome Alignment of Spencer

**Figure 3:**
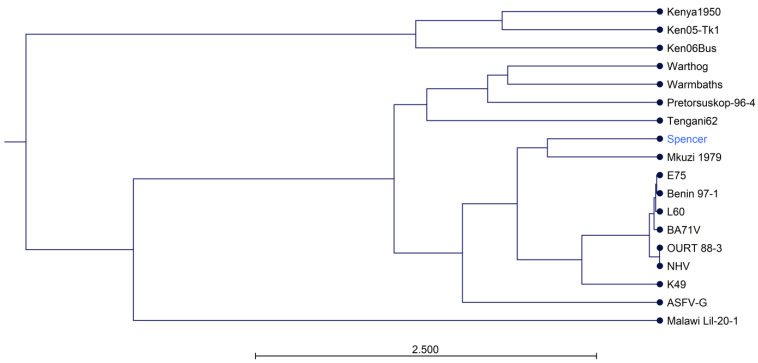
Phylogenetic tree constructed using the UPGMA method showing the relationships based on average nucleotide identity between Spencer and historic isolates. Scale indicates branch length.

The full-length genome of Spencer was aligned to 16 historic genomes. Based on average nucleotide percent identity, Spencer was most similar to Mkuzi1979 (98.35%). Spencer and Mkuzi1979 formed a clade that was a sister group to the genotype 1 clade composed of K49, BA71V, E75, L60, Benin 97-1, NHV, and OURT-883 (Figure 3). Georgia 2007/1 forms its own clade, which is an outgroup of the clade composed of genotype 1 isolates, Mkuzi1979, and Spencer.

**Figure 4:**
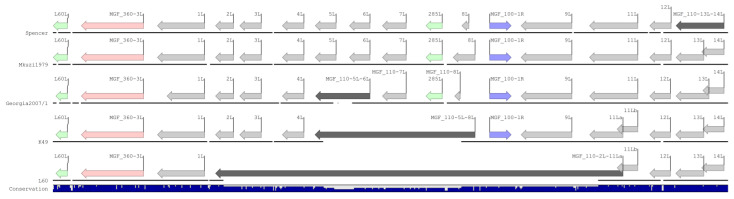
MGF 110 coding region of Spencer, Mkuzi1979, Georgia 2007/1, K49, and L60Mkuzi1979. Arrows indicate the coding direction of predicted ORFs belonging to MGF_110 (gray), MGF_100 (blue), MGF_360 (peach), or non-MGF (green) families. Dark gray ORFs indicate MGF_110 fusion proteins. Genomic deletions are represented by the absence of a black line. Royal blue bar graph beneath the figure indicates nucleotide conservation.

The alignment of the genomes revealed significant differences within the left variable region of the genome (Figure 4). As previously described, L60 and Benin 97/1 contain a deletion within this region that results in the fusion of MGF_110-11L and MGF_110-2L and the loss of 285L, MGF_100-1R, and MGF_110-3L through MGF_360-9L [14]. For simplicity, Benin97/1 was omitted from the figure. K49 has a smaller deletion that results in the fusion of MGF_110-8L and MGF_110-5L and the loss of 285L and MGF_110-5L through MGF_110-7L. Conversely, Spencer is more like Mkuzi1979 and Georgia 2007/1 in that it does not contain a deletion in this region, though Georgia 2007/1 does contain a roughly 320-nucleotide deletion that results in the fusion of MGF_110-5L and MGF_110-6L. Further, downstream of the deletion, within the MGF_110-14L ORF, is a highly variable homopolymer consisting of 17 (Georgia 2007/1), 16 (Spencer), 11 (K49), or 9 (Mkuzi1979 and L60) cytosines. Unlike the examined genomes, Spencer is predicted to encode a single ORF consisting of a fusion between MGF_110-13 and MGF_110-14-L. Accordingly, the LVR encoded by Spencer is more similar to Mkuzi1979 and Georgia compared to either of the genotype 1 isolates.

### 3.3. Analysis of Identical and Missing ORFs

**Figure 5:**
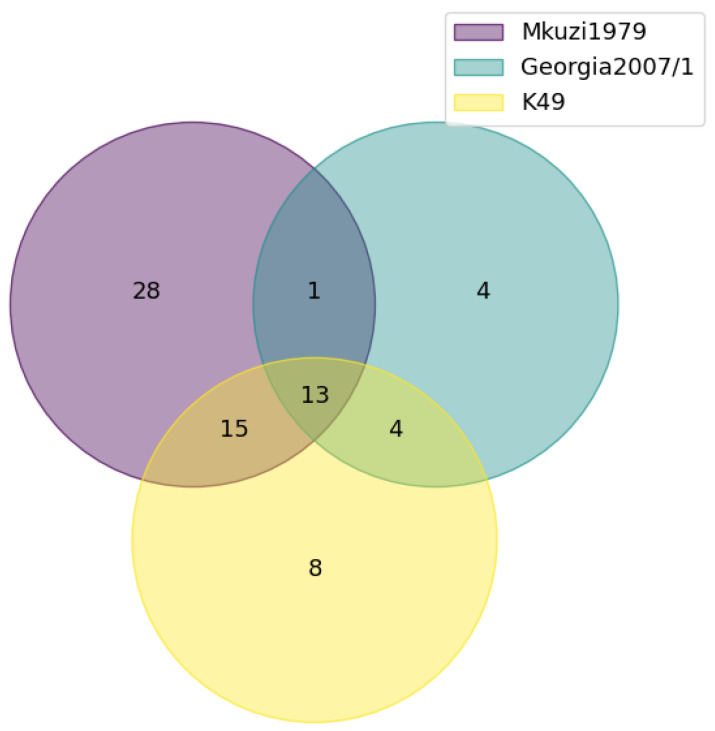
Venn diagram indicating the number of identical genes predicted to be encoded by Mkuzi1979, Georgia 2007/1, and K49 as compared to Spencer.

Spencer was annotated and predicted to encode 166 ORFs. The predicted amino acid sequences of the ORFs encoded by Spencer were aligned to their homologs encoded by Georgia 2007/1, Mkuzi1979, and K49 (Supplemental Table S1). Based on percent identity, there were thirteen ORFs (I8L, D1133L, C62L, B66L, E111R, A179L, B125R, B385R, B119L, H124R, A104R, C315R, and D250R) that were identical between all four isolates (Figure 5). Exclusively, there were twenty-eight ORFs encoded by Spencer that were 100% homologous to Mkuzi1979 (EP296R, I9R, MGF_360-15R, G1340L, L60L, I73R, H108R, MGF_100-3L, I177L, D345L, ACD_01870, ACD_00270, I215L, MGF_360-12L, MGF_360-3L, MGF_360-16R, O61R, MGF_360-18R, A224L, I329L, K78R, I7L, E66L, E146L, MGF_505-3R, MGF_360-13L, E301R, and D129L), eight ORFs that were 100% homologous to K49 (B175L, B646L, EP153R, D117L, I267L, DP96R, NP419L, and F165R), and four ORFs that were 100% homologous to Georgia 2007/1 (C129R, H171R, C257L, and B117L). Mkuzi1979 and K49 shared an additional fifteen ORFs (CP80R, I243L, F317L, E120R, E184L, B354L, D339L, H339R, EP1242L, K196R, B263R, MGF_360-14L, ACD_00600, A137R, and S273R) that were 100% homologous to Spencer, while Mkuzi1979 and Georgia 2007/1 shared an additional ORF (DP79L) that was 100% homologous to Spencer. Lastly, K49 and Georgia 2007/1 shared four ORFs (K145R, C122R, C717R, and F334L) that were 100% homologous to Spencer.

We also examined ORFs predicted in K49, Mkuzi1979, and Georgia 2007/1 that were absent in Spencer. Unlike Spencer, K49 encoded an additional copy of X69R, and Mkuzi1979 encoded an additional copy of I10L. At the 3′ ITR, Spencer is similar to K49 and L60 in that it is also missing MGF_360-21R. The Illumina reads mapped to the Georgia 2007/1 failed to align to this region, indicating that the missing MGF_360-21R was not the result of an aberrant assembly at the far end of the genome. Accordingly, an examination of the predicted ORFs encoded by Spencer indicates that some ORFs are identical to genotype 1 and genotype 2 isolates.

### 3.4. Analysis of Non-Identical ORFs

The remaining 61 non-hypothetical and non-MGF ORFs were aligned, and genetic distance and its variance between sequences, as compared to the predicted ORF encoded in Spencer, were calculated using the Jukes–Cantor model (Supplemental Table S1). Twenty-one ORFs (285L, A240L, A859L, B169L, B407L, B475L, B962L, C962R, E183L, EP364R, EP424R, F1055L, F778R, H233R, I10L, K421R, KP177R, L83L, M448R, NP1450L, and P1192R) were most closely related to Mkuzi1979; twenty ORFs (A151R, A238L, B602L, CP123L, CP204L, DP238L, DP96R, EP152R, EP153R, EP402R, H240R, H359L, I196L, K205R, L11L, M1249L, QP383R, QP509L, R298L, and X69R_1) were most closely related to L60 and/or K49; and two ORFs (E165 and E199L) were most similar to Georgia 2007/1. The following parenthetical genomes had identical distances and were most similar to Spencer, as compared to the other genomes: CP2475L and Q706L (Georgia 2007/1 and K49); C475L (Georgia 2007/1, L60, and Mkuzi1979); B318L (Georgia 2007/1 and Mkuzi); C84L and CP312R (Georgia 2007/1, Mkuzi1979, and K49); B438L, CP530R, DP71L, and EP84R (K49 and Mkuzi1979); C147L, E248R, G1211R, and I226R (K49, Mkuzi1979, and L60); and NP868R and O174L (Mkuzi1979 and L60). D205 and E423R had identical distances in Georgia 2007/1, K49, L60, and Mkuzi1979. Accordingly, an examination of the predicted ORFs encoded by Spencer indicates that some ORFs are more identical to genotype 1 or genotype 2 isolates.

## 4. Conclusions

In conclusion, the historical isolate Spencer isolated in 1951 from South Africa is a unique ASFV strain and may provide clues into ASFV evolution. It is possible that the ASFV isolate Spencer sequenced here could be a remnant of an ancestral strain of genotype 1 and genotype 2 isolates or a transitional strain signaling the evolution from genotype 1 to genotype 2. However, we still lack enough sequences of historical ASFV isolates to have a true understanding of how the ASFV evolved into different unique isolates over history, although this Spencer strain could be one of the first clues into the evolution of different historical isolates of the ASFV. Recently, there have been some reports of new hybrid genotype 1 and genotype 2 strains surfacing in Asia; however, these strains are very different from Spencer, where the genotype I strain most closely matching is from a historical genotype I vaccine that was used in Portugal, and the genotype II is from the current pandemic strain in Asia.

## Data Availability

Genome sequences have been deposited in GenBank under accession no. PP828951. Raw data for this project can be found in BioProject accession no. PRJNA1113662.

## Supplementary Materials

The following supporting information can be downloaded at https://www.mdpi.com/article/10.3390/v16081175/s1: Table S1: Spencer matrices.
